# Dendrites contribute to the gradient of intrinsic timescales encompassing cortical and subcortical brain networks

**DOI:** 10.3389/fncel.2024.1404605

**Published:** 2024-09-06

**Authors:** Kaichao Wu, Leonardo L. Gollo

**Affiliations:** Brain Networks and Modelling Laboratory, School of Psychological Sciences, and Monash Biomedical Imaging, The Turner Institute for Brain and Mental Health, Monash University, Melbourne, VIC, Australia

**Keywords:** intrinsic timescales, dendritic morphology, neuronal dynamics, functional networks, anatomical hierarchy

## Abstract

**Introduction:**

Cytoarchitectonic studies have uncovered a correlation between higher levels of cortical hierarchy and reduced dendritic size. This hierarchical organization extends to the brain's timescales, revealing longer intrinsic timescales at higher hierarchical levels. However, estimating the contribution of single-neuron dendritic morphology to the hierarchy of timescales, which is typically characterized at a macroscopic level, remains challenging.

**Method:**

Here we mapped the intrinsic timescales of six functional networks using functional magnetic resonance imaging (fMRI) data, and characterized the influence of neuronal dendritic size on intrinsic timescales of brain regions, utilizing a multicompartmental neuronal modeling approach based on digitally reconstructed neurons.

**Results:**

The fMRI results revealed a hierarchy of intrinsic timescales encompassing both cortical and subcortical brain regions. The neuronal modeling indicated that neurons with larger dendritic structures exhibit shorter intrinsic timescales. Together these findings highlight the contribution of dendrites at the neuronal level to the hierarchy of intrinsic timescales at the whole-brain level.

**Discussion:**

This study sheds light on the intricate relationship between neuronal structure, cytoarchitectonic maps, and the hierarchy of timescales in the brain.

## 1 Introduction

The human brain continually integrates and processes multiscale external inputs resorting to its intrinsic neural timescales (INT) (Murray et al., 2014; Hasson et al., 2015; Chaudhuri et al., 2015; Gollo et al., 2015, 2017; Farzan et al., 2017; Wasmuht et al., 2018; Liégeois et al., 2019; Gollo, 2019; Deco et al., 2019; Wolff et al., 2022). Depending on the specific brain functions and development stage, different brain regions have distinct intrinsic timescales over which they integrate information (Truzzi and Cusack, 2023). Typically, the range of INT goes from shorter in lower-order unimodal sensory-motor regions to longer in high-order transmodal regions (Kiebel et al., 2008). This gradient of INT can be observed across multiple neuroimaging modalities, including electroencephalography (EEG) (Smith et al., 2022; Wolman et al., 2023), magnetoencephalography (MEG) (Demirtaş et al., 2019; Golesorkhi et al., 2021), and functional magnetic resonance imaging (fMRI) (Burt et al., 2018; Raut et al., 2020a; Watanabe et al., 2019). Among the different modalities, fMRI stands out for its exceptional spatial resolution. The hierarchy of INT is a common finding on either small (Raut et al., 2020a; Wasmuht et al., 2018) or large-scale fMRI datasets (Burt et al., 2018; Ito et al., 2020). On the cortical level, sensory and motor regions/networks, the unimodal regions, typically display shorter INT, whereas higher-order networks, also know as the transmodal regions (Wolff et al., 2022; Raut et al., 2020a; Huntenburg et al., 2018), like the central-executive networks (CEN), dorsal attention networks (DAN), and default-mode network (DMN) tend to exhibit longer INT (Demirtaş et al., 2019; Golesorkhi et al., 2021; Ito et al., 2020). This temporal hierarchy extends to the subcortical level as well. Within subcortical regions such as the thalamus, cerebellum, striatum, and hippocampus, there exists a gradient INT. The thalamus, for instance, exhibits relatively short INT, enabling rapid transmission of incoming external sensory information to the cortex for further processing. Conversely, the hippocampus exhibits some of the longest intrinsic neural timescales in the brain, facilitating the integration and storage of information over extended periods to support the consolidation of episodic and spatial memories (Raut et al., 2020a).

**Figure 1:**
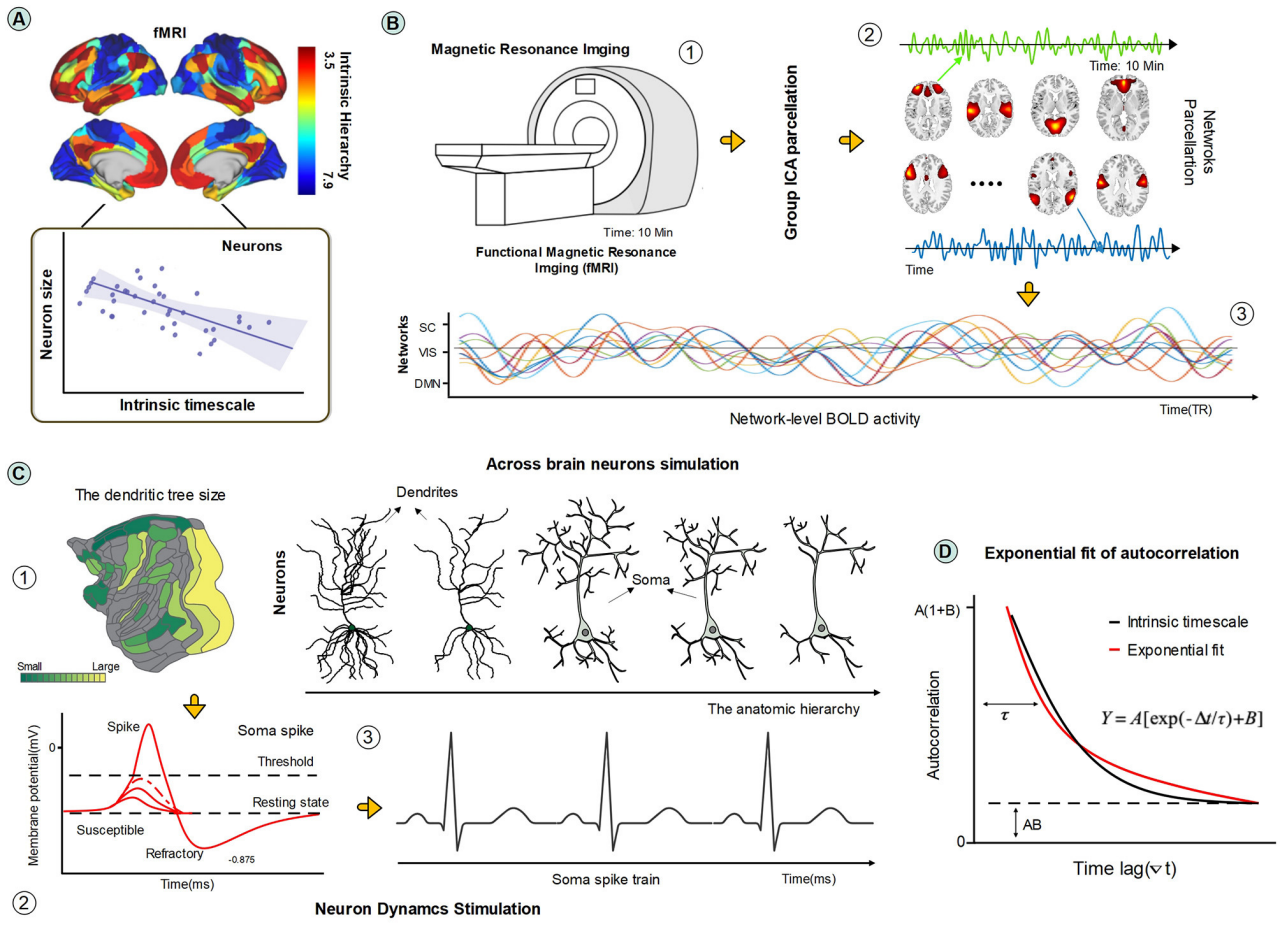
**(A)** Study design. Brain regions display heterogeneous intrinsic timescales that mirror a gradient reflecting the cortical hierarchy. Cytoarchitectonic findings indicate a link between anatomical level and dendritic size (Hilgetag et al., 2019). We hypothesize the presence of an association between dendritic size and INT. The brain map was adapted from Wolff et al. (2022). **(B)** Three steps for whole-brain (encompassing subcortical and cortical regions) functional network identification: with the functional image obtained (step 1), the group ICA method was applied to obtain the brain parcellation of functional networks (step 2), and then the corresponding timeseries of generated ICA components were extracted to represent the network-level neural activity (step 3). **(C)** Cytoarchitectonic mappings suggest that neurons with larger dendrites are situated at lower hierarchical levels, the macroscopic brain map was adapted from Hilgetag et al. (2019). Neuronal activity was simulated using multicompartmental models featuring excitable dynamics (SIRS) for neurons exhibiting varying degrees of dendritic integrity, thereby represent neurons positioned at distinct levels of the anatomical hierarchy. **(D)** The INT was computed for the BOLD fMRI time-series of brain networks and for the neuronal time-series from the somatic compartment. The INT was computed based on the decay properties of the autocorrelation function (see Methods).

The gradient of INT encapsulated by the principle of a hierarchy of timescales in the brain permeates the different spatial scales from the single-neuron level (Murray et al., 2014) to the whole brain (Kiebel et al., 2008; Raut et al., 2020a). A close relationship exists between INT and functional connectivity patterns of the brain regions (Northoff et al., 2010; Northoff and Gomez-Pilar, 2021), as shown in Figure 1A. These connections play a significant role in shaping the brain's state, behaviors, and cognition (Van Den Heuvel and Pol, 2010; Power et al., 2011; Yeo et al., 2011). They form a gradient of intrinsic timescales, with an increased contribution of slow fluctuations at higher levels of the cortical hierarchy (Raut et al., 2020b). More generally, the anatomical hierarchy in the brain is regarded as a basis of the variation of INT across brain regions (Murray et al., 2014; Fallon et al., 2020) . This idea that anatomical hierarchy determines the region's temporal dynamics can extend to the brain's core-peripheral network organization. Highly connected hub regions that form a rich club (Van Den Heuvel and Sporns, 2011), typically exhibit slower INT compared to peripheral areas located in sensorimotor systems (Gollo et al., 2015; Chaudhuri et al., 2015). Beyond the level of cortical regions, the structure and local neuronal connectivity (connection strength and coupling pattern) also show relevance to the gradient of INT (Chaudhuri et al., 2015; Runyan et al., 2017). Regions with longer intrinsic timescales contained neurons with stronger local excitatory connections (Cohen and Kohn, 2011; Wasmuht et al., 2018). Furthermore, it has been proposed that the diversity of neuronal function and timescales stems from variations in the densities of dendritic spines (Elston, 2003; Cavanagh et al., 2020).

A growing body of literature has indicated a strong connection between the structural (or anatomical) hierarchical level of the brain and fundamental features of its dynamics (Kiebel et al., 2008; Cocchi et al., 2016; D'Souza et al., 2016; Burt et al., 2018), observable from the microscopic scale of single neurons to the indirect large-scale dynamics captured by blood oxygenation level-dependent (BOLD) signals. Yet, the precise mechanisms underlying this relationship remain largely elusive. Cytoarchitectonic studies have revealed a significant correlation between the size of neuronal dendrites and the anatomical hierarchical level to which neurons belong (Beul and Hilgetag, 2019; Hilgetag et al., 2019)(Figure 1C). Typically, neurons situated at higher anatomical levels exhibit smaller dendritic sizes in their pyramidal neurons. This correlation motivates the question: What is the role of neuronal morphology in shaping the gradient of intrinsic timescales across the brain?

Here we investigate the whole-brain dynamics, focusing on the subcortical-cortical INT hierarchy and the contribution of the neuronal dendritic size to such a gradient of INT. Specifically, we examined resting-state functional MRI data from a cohort of 34 healthy young adults (aged 19 to 22) to investigate the gradient of INT across both subcortical and cortical brain regions. Utilizing group-independent component analysis (ICA), we extract the regional activity, and measure the network-level INT. To investigate the neuronal contribution behind the gradient of INT, we explore the dynamics of a multi-compartmental neuron model (Kirch and Gollo, 2020), endowed with a progressive pruning process (Kirch and Gollo, 2021) to estimate the impact of dendritic size on INT. Consistent with cytoarchitectonic findings, we hypothesize that the neuronal INT would increase as neurons undergo progressive pruning.

## 2 Materials and methods

### 2.1 Subcortical and cortical functional network dynamics

Figure 1B shows the three steps for the identification of brain functional networks dynamics : (1) the fMRI scans acquisition and processing; (2) Group ICA for Network Parcellation; and (3) Post-processing for BOLD timeseries extraction. These three steps are described in detail next.

#### 2.1.1 fMRI acquisition and preprocessing

Resting-state fMRI scans were obtained from the available public dataset of the University of North Carolina samples at Greensboro (Wahlheim et al., 2022; Wu et al., 2023b). The participants were 34 healthy young adults (18–32 years old, mean: 22.21, SD: 3.65). The functional MRI was acquired with an echo-planar imaging sequence: 32 slices with 4.0 mm thickness and no skip, time of echo = 30 ms; time of repetition (TR) = 2000 ms; flip angle = 70°, field of view = 220 mm, matrix size = 74 × 74 × 32 voxels. Each fMRI scan lasted for 10 min, comprising 300 volumes. Additional details about the raw fMRI data can be found in Wahlheim et al. (2022).

To generate a steady blood oxygenation level-dependent activity signal, the first five volumes of each scan were discarded to allow for magnetic stability. Similar to the previous studies (Wu et al., 2024b, 2023a), the functional data was then processed with the following steps: (1) Realignment to correct head motion for verification details; (2) Slice time correction. (3) Outlier identification (The volumes at the time point would be regarded as outliers and removed if the signal value is three standard deviations beyond the mean global signal for the entire run or if the head motion exceeded 0.5 mm in any direction). (4) Normalization (normalize to 3 mm MNI space using a template from the SPM software package; Friston, 2003). (5) Spatial smoothing with a Gaussian kernel of 8 mm full-width at half-maximum (FWHM).

#### 2.1.2 Group ICA for Network Parcellation

A spatial group ICA was performed on the preprocessed and denoised BOLD signal using the Group ICA of FMRI Toolbox (GIFT) infomax algorithm. Specifically, high-model order ICA with a set of 100 components was obtained, comprising resting-state brain networks spanning cerebral cortical and subcortical regions. The configuration for the group ICA algorithm was developed according to the detailed description provided by (Wu et al., 2023b; Salman et al., 2019). In particular, a two-stage Principal Component Algorithm (PCA) method was first adapted to preserve the components that account for the most variance. The top 120 principal components (PCs) of all participants obtained in the first stage were concatenated across time and then further reduced to 100 in the second stage. Then, the infomax algorithm was used with 20 repeats to find steady independent components (ICs). After back reconstruction, the participant-specific spatial maps and corresponding time courses were obtained.

Three methods were employed to detect activated potential functional networks from the IC reservoir. (1) The spatial activation maps from the ICs were visually inspected to identify if they matched the large-scale functional network locations from previous studies and to make sure they were located at gray matter volumes. (2) The multiple regression method was used to produce the weight of ICs whose spatial pattern matches with the existing functional network template. The weights were used to rank the list, and the functional network that matched the most was selected. (3) The power spectrum of the ICs was checked to see if it follows a low-frequency peak and a high-frequency steady pattern (the time courses of ICs are characterized by high dynamic range). Those ICs that located cerebrospinal fluid and ICs whose highest regression weights were significantly low and whose power-spectrum curves were different (e.g., a clear peak of the wave at high frequency) were discarded.

#### 2.1.3 Post-processing for BOLD timeseries extraction

After removing noise-related components, the time courses of retained components were triple detrended and despiked (Wu et al., 2024a). The motion parameters and global average were regressed for postprocessing, and a band-pass filter was applied (0.023-0.1 Hz). These actions ensure artifact noise can be largely eliminated and has minimal impact on the further signal analysis.

### 2.2 Neuronal modeling

To simulate the dynamics of neurons with detailed dendritic structure, we used digitally reconstructed neurons obtained from the neuromorpho database (Ascoli et al., 2007). These neurons were explored using a pruning algorithm that iteratively removes the most distal dendritic compartments (Kirch and Gollo, 2021). For the different neuronal structures, from the intact neuron to a highly pruned dendritic tree, the neuronal dynamics was simulated using an active dendritic model in which each compartment generates dendritic spikes (Gollo et al., 2009, 2012; Kirch and Gollo, 2020, 2021). These steps can be seen in Figure 1C and are described in more detail next.

#### 2.2.1 Prototype neuron

**Figure 2:**
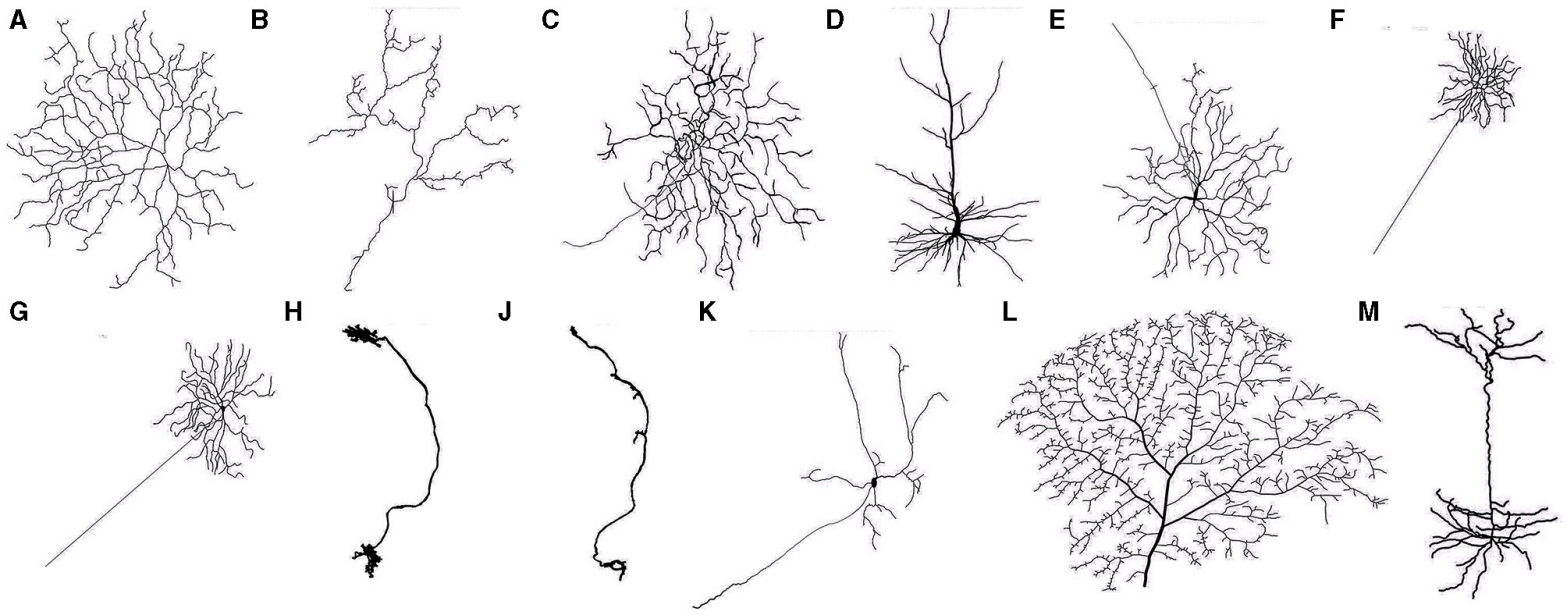
Neurons **(A–M)** (see details of neuronal morphology in Table 1).

The first step of calculating the neuron dynamics is to stimulate and construct the neurons with varying levels of dendritic integrity. It resorts to initial neurons with rich morphological shapes, which we call prototype neurons, and this process is implemented by iteratively pruning their terminal dendritic compartments. Specifically, the prototype neurons were taken from the NeuroMorpho database (Ascoli et al., 2007). This database contains thousands of neurons with detailed dendritic structure, which can be used to simulate multicompartmental neurons. To avoid the bias provided by the prototype neurons' details (i.e., the species, human or animal), location (i.e., the brain regions), and topology (i.e., spatial information), 13 high-quality prototype neurons were randomly selected across 6 species and 5 brain regions. The morphology of these prototype neurons has been presented in Figure 2, and their details, including the number of their compartments and bifurcations, can be seen in Table 1.

**Table 1:** List of Prototype neurons taken from NeuroMorpho.Org.

	**Name**	**Species**	**Regions**	**Num. of branches**	**Num. of compartments**	**Num. of bifurcations**	**References**
A	Mice2013	Mouse	Retina	5	10,175	175	Trakhtenberg et al., 2017
B	057	Fish	Retina	3	3,628	42	Pushchin, 2017
C	Shi2013_Fig8_O	Mouse	Retina	4	2,749	238	Shi et al., 2013
D	197-1-6mt	Human	Neocortex	10	422	106	Jacobs et al., 2018
E	LY14-RGC11	Rat	Retina	6	1,216	320	Rodger et al., 2012
F	20190517_C10_WT	Mouse	Retina	3	1,812	87	Werginz et al., 2020
G	20170907_S2W1C1	Mouse	Retina	6	2,777	88	Raghuram et al., 2019
H	160510_lmage7	Zebrafish	Neocortex	2	1,579	136	Kunst et al., 2019
I	170221_11_1	Zebrafish	Central nervous system	2	422	30	Kunst et al., 2019
J	Gau-D27R-3	Pouched lamprey	Optic lobe	4	713	38	Fletcher et al., 2014
K	Badea2011Fig2Cj	Mouse	Retina	2	1,443	184	Badea, 2011
L	dHSE_05l	Drosophila melanogaster	Optic lobe	2	4658	1525	Cuntz et al., 2013
M	14,044_L5_PYr_PM	Mouse	Neocortex	7	1,579	29	D'Souza et al., 2016

#### 2.2.2 Neuron pruning

Previous cytoarchitectonic findings suggested that along the spatial cortical gradients, the higher the anatomical level, the smaller the dendritic size of their pyramidal neuron tends to be (Beul and Hilgetag, 2019; Hilgetag et al., 2019). Hence, we utilized the proposed pruning algorithm with the prototype neurons to simulate neurons with variable dendritic sizes. Specifically, the pruning algorithm used an iterative approach to progressively remove the most terminal compartments (Kirch and Gollo, 2021). At each iteration, the terminus was detected and then removed until the entire dendritic tree was eliminated (see Figure 1C for an illustration of the pruning process). In our experiments, the maximum iteration step explored was 80. The neuronal morphologies obtained in intervals of 10 pruning iterations were explored. Consequently, for each prototype neuron, nine different dendrites were studied, resulting in a total of 117 different neuronal structures.

#### 2.2.3 Neuronal dynamics

The neuronal dynamics was simulated and the activity at the soma was recorded for subsequent analyses. Specifically, we adapted a synchronous susceptible–infected (active)–refractory–susceptive model (SIRS) to simulate the dynamics of each active dendritic compartment, and particular attention was given to the somatic spiking activity (Gollo et al., 2009, 2012, 2013; Kirch and Gollo, 2020, 2021). Each excitable compartment can be in the following 3 states: susceptible, active (spike), and refectory. Dendritic compartments become active, generating dendritic spikes, either by external synaptic input or by the propagation of activity from neighboring dendritic compartments with a probability equals *p*_λ_. As full spatial distributions of ion channels governing the dynamics of dendritic compartments remains largely unknown, here we considered the simplest case of *p*_λ_ = 0.97, which was homogeneous across the entire dendritic tree. In our experiments, the external synaptic stimuli were modeled as a stochastic process, and the active rate was a Poisson process with rate *r* = 0.03. Active compartments become refractory at the next time step (δ*t* = 1ms). The refractory compartments become susceptible after a period of δ*r* = 8ms. Unless activated externally via synapses or by propagation from an active neighbor, dendritic compartments remain in a susceptible state. For each neuron, the simulations were run for a duration of 100,000 time steps (1000s). The soma was modeled as a single-compartment that has the same properties of other dendritic compartments.

This model dynamics representing the activity of thousands of dendritic compartments was firstly proposed by Gollo et al. (2009) to show that neurons with large active dendrites, typical of somatosensory regions (Beul and Hilgetag, 2019), can significantly increase the dynamic range of neurons, allowing them to respond to a wide range of input levels. The model was further analyzed using the excitable-wave mean-field approximation (Gollo et al., 2012), and its generalized form (Gollo et al., 2013) to gain insights into the role homogeneous and non-stereotyped dendritic spikes caused by non-homogeneous distribution of ion channels. Additionally, this model (Gollo et al., 2009, 2012) was able to reproduce not only standard sigmoid response functions but also various types of complex response functions, such as double sigmoid curves obtained from the retinal ganglion cells of the mouse (Deans et al., 2002). More recently, this neuronal model has been further refined (Kirch and Gollo, 2020, 2021) to more accurately reflect real biological processes by using the real morphology of neurons obtained from digital reconstructions available in the NeuroMorpho database (Ascoli et al., 2007).

### 2.3 Intrinsic neural timescales

The intrinsic neural timescales (INT) were computed for brain networks and for single-neuron dynamics using two complementary computational methods based on the autocorrelation function. These methods accommodate data with different temporal resolutions. In most electrophysiological studies, which involve fast dynamics, the intrinsic neural timescales were calculated as the timescale of an exponential decay coefficient fitted to the autocorrelation curve (Runyan et al., 2017; Murray et al., 2014; Bernacchia et al., 2011). The INT obtained from this fitting method is called (*INT*_*f*_). For resting-state fMRI, which has lower temporal resolution, the intrinsic neural timescales were computed as the area under the autocorrelation curve to mitigate the adverse effects of low sampling rates (Watanabe et al., 2019; Xie et al., 2023; Raut et al., 2020a). The INT obtained from the area under the curve method is called (*INT*_*a*_).

Specifically, *INT*_*f*_ was defined as the decay coefficient of the autocorrelation of the BOLD fMRI time-series and soma timeseries(see Figure 1D). The autocorrelation function (ACF) of the timeseries from the corresponding ICA components and soma dynamics were calculated with the following formula:


(1) $$\begin{array}{l} AFC_{k}=\frac{\sum_{t=k+1}^{T}\left(y_{t}-ȳ\right)\left(y_{t-k}-ȳ\right)}{\sum_{t=1}^{T}{\left(y_{t}-ȳ\right)}^{2}}. \end{array}$$

For brain regions, *y* denotes the rs-fMRI BOLD timeseries, ȳ is the mean value across time points, *t* is the length of time bins which is the time of repetition (TR = 2000 ms) of MRI scan, and *T* is the number of time points (295 in the experiment). For the single-neuron model, *y* denotes the somatic timeseries from stimulated neuron dynamics, ȳ is the mean value across stimulation steps, and *T* is the number of steps (100,000 s). *k* is the time lag. Then, we used autoregressive moving average (ARMA) models to fit the ACF curve (Olszowy et al., 2019; Truzzi and Cusack, 2023) and calculated the decay coefficient as the final measure of intrinsic timescales with the following equation:


(2) $$\begin{array}{lll} Y & = & A\left[exp\left(-△t/\tau \right)+B\right], \end{array}$$

where *A* is the scaling coefficient, and *B* is the offset, which also represents the asymptotic level of autocorrelation, and the *INT*_*f*_ = τ. For both the network and single neuron levels, a maximum of 25 lag intervals was used to fit the decay curve.

In addition, to avoid the bias brought by the definition of INT in resting-state fMRI signals, the INT at the brain network level was also calculated by the magnitude of ACF (Watanabe et al., 2019; Xie et al., 2023; Raut et al., 2020a). In this case, *INT*_*a*_ is defined by the area under the curve (AUC) of the initial ACF curve until it reaches a zero value:


(3) $$\begin{array}{l} INT_{a}=TR\cdot \overset{N}{\underset{k=1}{\sum }}ACF_{k}, \end{array}$$

where TR is the time of repetition, and *N* is the minimum time lag in which the autocorrelation attains a value less than or equal to zero.

Both *INT*_*a*_ and *INT*_*f*_ were computed for all ICA components and an intrinsic timescales map of the whole brain was computed for each participant. The dynamics of the single neurons, shown in Table 1, were computed for the different levels of dendritic pruning using *INT*_*f*_.

## 3 Results

To map the INT at the whole-brain level, the resting-state networks (RSN) were identified from function imaging using the group ICA method. The INT of neural activity corresponding to the RSN was then calculated across brain networks. To address the contribution of dendritic size, the digitally reconstructed compartment-based neurons were simulated, and their INT was computed. By investigating the association between the INT and neuron size, we analyze the contribution of neuronal dendrites to the gradient of intrinsic timescales that encompasses cortical and subcortical brain networks.

### 3.1 INT of brain functional networks

#### 3.1.1 The recognized resting state network

**Figure 3:**
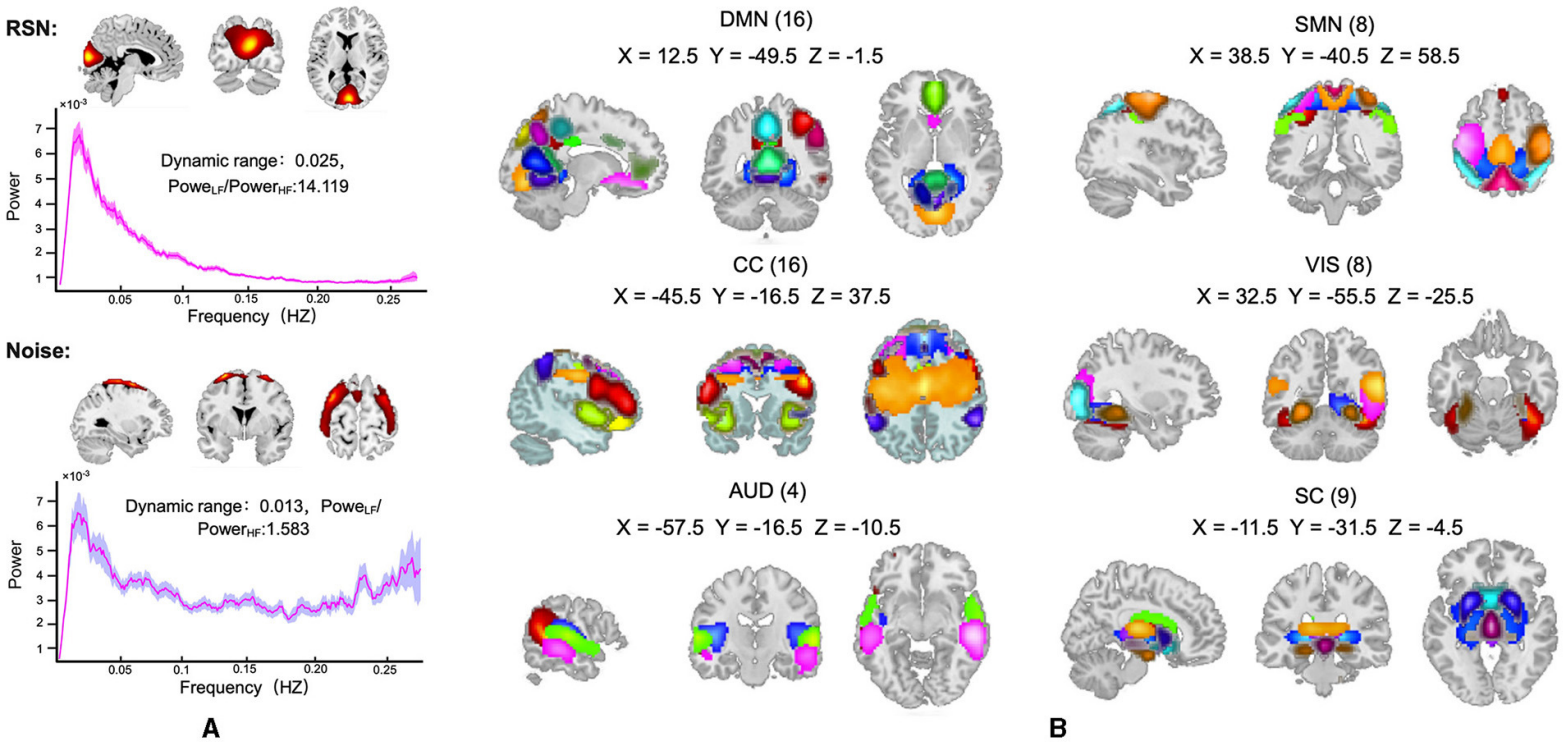
**(A)** An example of recognized RSN (top) and noise components (bottom), where the spatial map of components that represent the real brain activity should have clear boundaries and the corresponding power-frequency curve should peak at low frequency and decrease rapidly. **(B)** the spatial map of the 60 RSN components, which were assigned to six functional domains: SC, subcortical network; AUD, auditory network; VIS, visual network; SM, sensorimotor network; CC, cognitive control network; and DMN, default mode network.

Out of the 100 ICs identified by the group ICA, 40 ICs were identified as noise components and discarded. The remaining 60 components were assigned as RSN (an example of a clean signal component taken, and another example of a noise signal component discarded are presented in Figure 3). The 60 RSN components were assigned to six functional domains that have been widely studied for brain networks: subcortical network (SC), auditory network (AUD), visual network (VIS), sensorimotor network (SM), cognitive control network (CC), and default mode network (DMN). The spatial map of 60 RSNs and corresponding peak coordinates have been provided in the Supplementary Table 1 and Supplementary Figure 1.

#### 3.1.2 INT across functional networks

**Figure 4:**
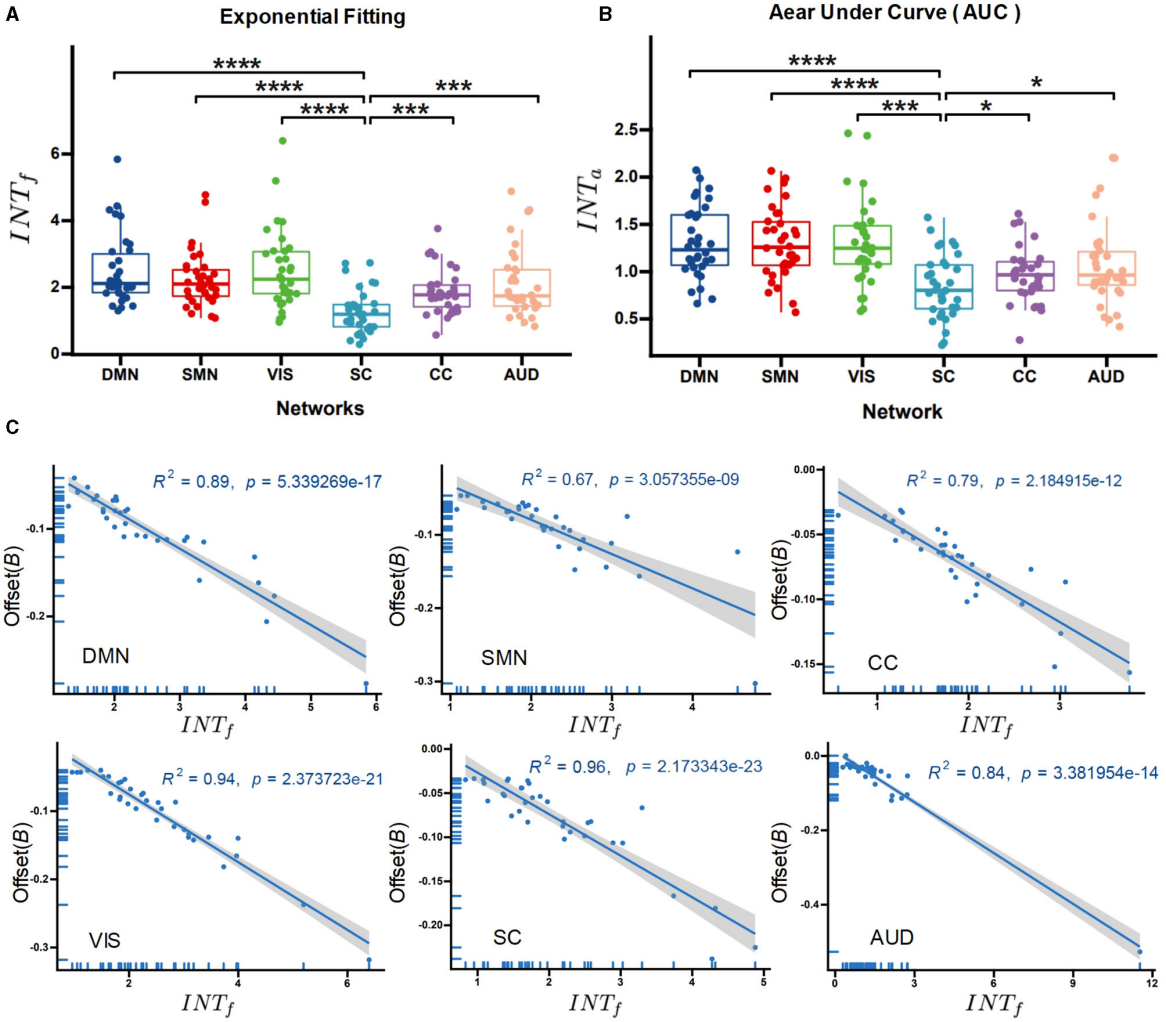
Between-network comparison of the intrinsic neural timescales. The exponential-fitting method (*INT*_*f*_) **(A)** and the area under the curve method (*INT*_*a*_) **(B)** for the different functional networks. **(C)** Regression analysis of the correlation between the offset of the exponential fitting and *INT*_*f*_. ^****^p < 0.0001 (FDR-corrected); ^***^p < 0.001 (FDR-corrected); ^*^p < 0.05 (FDR-corrected).

We utilized a one-way ANOVA followed by *post-hoc two-sample t-tests* to compare the intrinsic timescales across functional networks. The results (shown in Figure 4A) indicate significant group effects on the functional networks (*F* = 14.612, p < 0.001) for *INT*_*f*_. *Post-hoc t-test* shows that the subcortical network exhibited the lowest intrinsic timescales compared to other high-level cortical networks (*t*_*SC*−*DMN*_ = 3.32, p < 0.001; *t*_*SC*−*VIS*_ = 2.86 p < 0.001; *t*_*SC*−*SMN*_ = 3.76, p < 0.001; *t*_*SC*−*CC*_ = 2.78 p < 0.05, *t*_*SC*−*AUD*_ = 2.32, p < 0.05, FDR corrected). The *INT*_*a*_ method indicates similar results (*t*_*SC*−*DMN*_ = 4.12, p < 0.001; *t*_*SC*−*VIS*_ = 4.86 p < 0.001; *t*_*SC*−*SMN*_ = 3.76, p < 0.001; *t*_*SC*−*CC*_ = 3.68 p < 0.05, *t*_*SC*−*AUD*_ = 2.12, p < 0.05, FDR corrected; Figure 4B), sustaining the findings that functional networks involved in high-level processes tend to have longer intrinsic timescales.

In addition, the correlation between the offset of exponential fitting and *INT*_*f*_ was calculated via regression analysis. It was found that *INT*_*f*_ negatively correlates with the autocorrelation offset for each functional network (Figure 4C, see the detailed results in Table 2). The offset measures the distance between the obtained timescales and the observation window, which can reflect the contribution of timescales to neural fluctuations (Murray et al., 2014).

**Table 2:** The correlation between the offset of exponential fitting and *INT*_*f*_ via a regression analysis.

**Brain functional network**	**Slope**	***R* ^2^**	**P**
DMN	-0.048	0.89	< 0.0001
SMN	-0.054	0.67	< 0.0001
VIS	-0.051	0.94	< 0.0001
CC	-0.048	0.79	< 0.0001
VIS	-0.047	0.94	< 0.0001
AUD	-0.032	0.84	< 0.0001
SC	-0.039	0.96	< 0.0001

#### 3.1.3 The hierarchy of INT from subcortical to cortical networks

**Figure 5:**
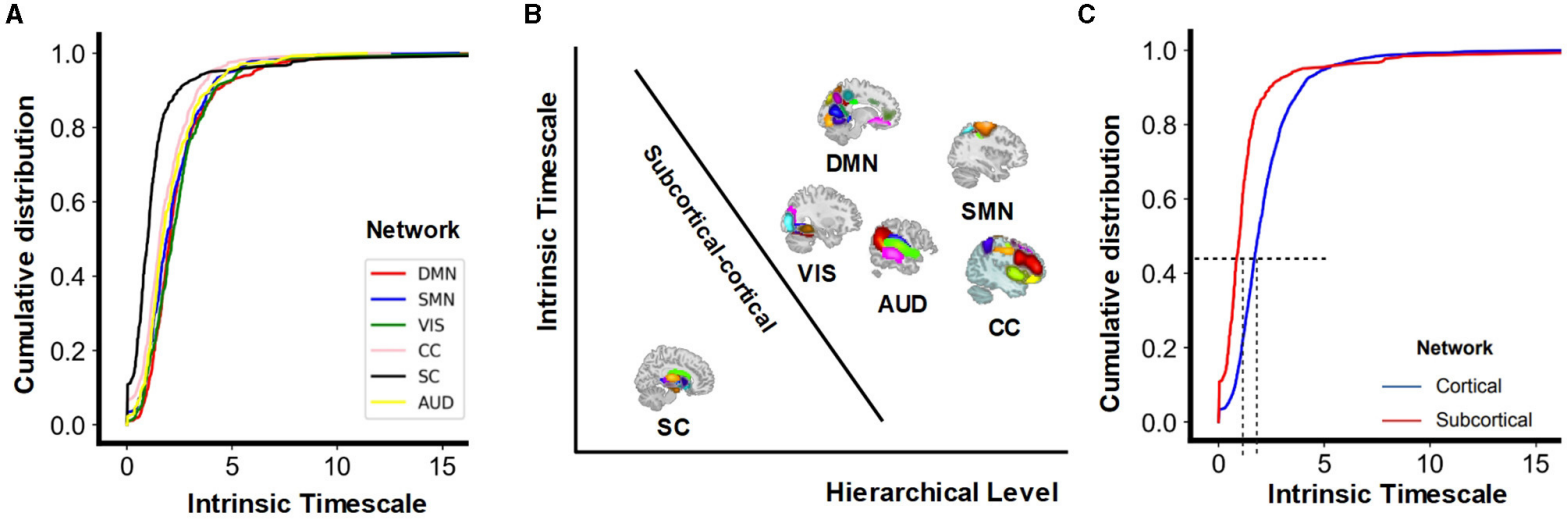
**(A)** Cumulative distribution of intrinsic timescales (*INT*_*f*_) for multiple networks. **(B)** Schematic distinction between the subcortical network at the lowest hierarchical level with shorter temporal integration, and cortical networks at higher hierarchical levels with longer temporal integration. The y-axis represents the length of the intrinsic timescales, and the x-axis represents the level of the hierarchy. The subcortical networks occupy a central position within the brain at lower hierarchical levels, whereas the cortical networks are situated more peripherally, at higher hierarchical levels. **(C)** Cumulative distribution of *INT*_*f*_ for the subcortical network and the combined cortical networks.

With the natural hierarchical and spatial gap between subcortical and cortical functional networks, we computed the cumulative distribution of *INT*_*f*_ of multiple brain networks. The cumulative distribution of intrinsic timescales shows significant differences across the function networks (*F* = 13.37, *p* < 0.0001). While with some specific probabilities, for example, 0.5, the VIS has the highest intrinsic timescales, DMN is the second, and then followed by the SMN and AUD. According to spatial location (the subcortical network is located more inside the brain). DMN, SMN, VIS, CC, and AUD are combined together into *cortical* networks. Results (Figure 5) show longer INT for cortical networks and shorter INT for the subcortical network (two-sample t-test: *t*_*cortical*−*subcortical*_ = 6.09, 95% CI [0.50, 096] *p* < 0.0001). This finding indicates a distinct temporal organization between the subcortical and cortical networks, implying that neural dynamics and information processing vary depending on their specialized functions.

### 3.2 INT of single neuron

#### 3.2.1 Neuron pruning and dynamics

**Figure 6:**
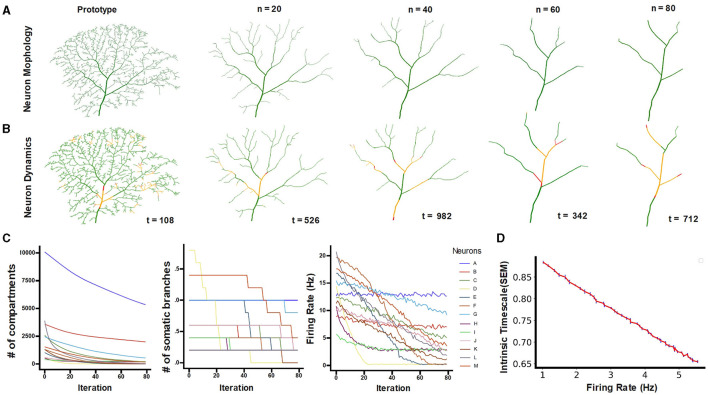
**(A)** The morphology of the prototype neuron “L” and the resulting pruned dendrites (at the number of pruning iterations n = 20, 40, 60, and 80). **(B)** Exemplary snapshots of the simulated neurons: red denotes spiking compartments, green denotes susceptible compartments, and yellow denotes refractory compartments. **(C)** Number of neuronal compartments, connections to the soma, and somatic firing rate as a function of the number of pruning iterations for the different neurons (as identified in Table 1). **(D)** The firing rate vs. intrinsic timescales accounts for longer intrinsic timescales of neurons with smaller dendrites. The blue error bars indicate the Standard Error of Mean (SEM) for 10 trials.

To obtain neurons with different levels of dendritic-tree integrity, a pruning algorithm was used to prototype neurons obtained from the NeuroMorpho database (Ascoli et al., 2007). Figure 6A presents an example of the morphology of a prototype neuron. As the pruning algorithm proceeds, the terminal dendritic compartments are progressively removed; as a consequence, the neuron integrity is reduced. The resulting morphology of a simulated neuron at iterations 20, 40, and 80 can be seen in Figure 6A respectively.

At each iteration, considering the dynamics of each compartment as an excitable unit (Kirch and Gollo, 2021), the neuronal dynamics were simulated. The tree-shaped neuron forms an excitable network, and the spiking activity of compartments determines the temporal evolution of activity and the corresponding somatic spikes. Figure 6B illustrates snapshots of neuronal activity at different time steps for neurons with different levels of dendritic integrity.

#### 3.2.2 The association between the INT and neuron size

As the pruning algorithm proceeds, the neuronal integrity is progressively affected, and the number of compartments and connections to the soma is reduced (Figure 6C). This pruning process impacts the neuronal dynamics, and causes a substantial reduction in the firing rate in most neurons (Figure 6C). To gain a deeper insight into how firing rate influences intrinsic timescales, we simulated the dynamics of neurons in the absence of dendrites, that is, at the last pruning iteration when all the neurons are identical and only the neuronal soma remains. For these punctual neuron models, we vary the rate of input (modeled as a Poisson process) that controls the firing rate of neurons and measure the intrinsic timescales. The results show that the intrinsic timescales decay linearly with the firing rate of the neuron (Figure 6D).

**Figure 7:**
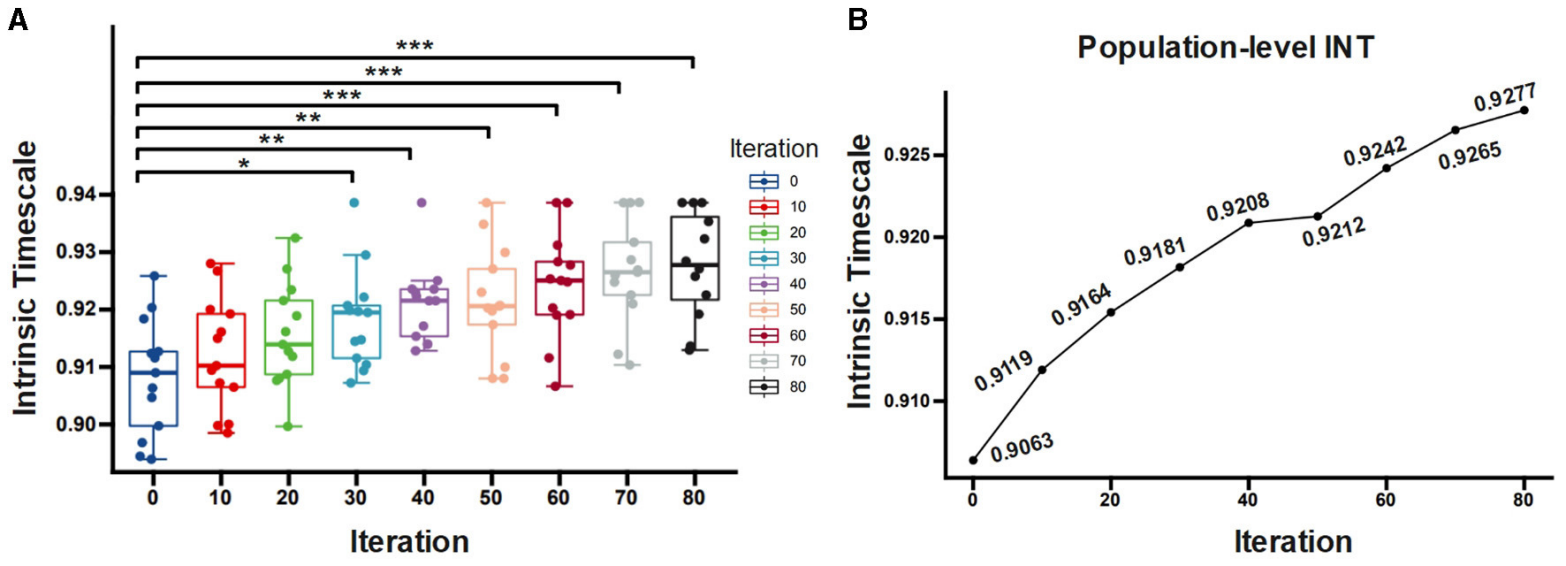
**(A)** Comparison of *INT*_*f*_ for neurons with different pruning iterations. **(B)** *INT*_*f*_ computed at the population level as a function of the number of pruning iterations. ^**^p < 0.01 (FDR-corrected); ^***^p < 0.001 (FDR-corrected); ^*^p < 0.05 (FDR-corrected).

We then examine the association between the INT and dendritic size. The statistical test (see Figure 7A) demonstrates that the prototype and pruned neuron have significantly different *INT*_*f*_ (F = 5.1639, p = 0.0001). Compared to the prototype neuron, the pruned neurons exhibit significant increased *INT*_*f*_ for iterations greater than 30 (*post-hoc t-test*, *t*_30_ = 2.639 *p* = 0.014; *t*_40_ = 3.443 *p* = 0.002; *t*_50_ = 3, 243 *p* = 0.003; *t*_60_ = 3.927 *p* = 0.0006; *t*_70_ = 4.472 *p* = 0.004; *t*_80_ = 4.776 *p* < 0.0001, FDR-corrected). The population-level results of *INT*_*f*_ calculation (the best fitting curve for all neurons at the same iteration) also reveal that as the pruning iteration progresses, *INT*_*f*_ increases. The prototype neuron exhibits the shortest *INT*_*f*_ of 0.9063, while the maximally pruned neuron displays the longest *INT*_*f*_ of 0.9277 (Figure 7B). The pruning algorithm reduces the size and complexity of the dendrites, which causes an alteration in the INT. Our results suggest an inverse relationship: neurons possessing larger dendrites exhibit shorter INT, while those with smaller dendrites display longer INT.

#### 3.2.3 Dendrites contribute to the hierarchy of INT

**Figure 8:**
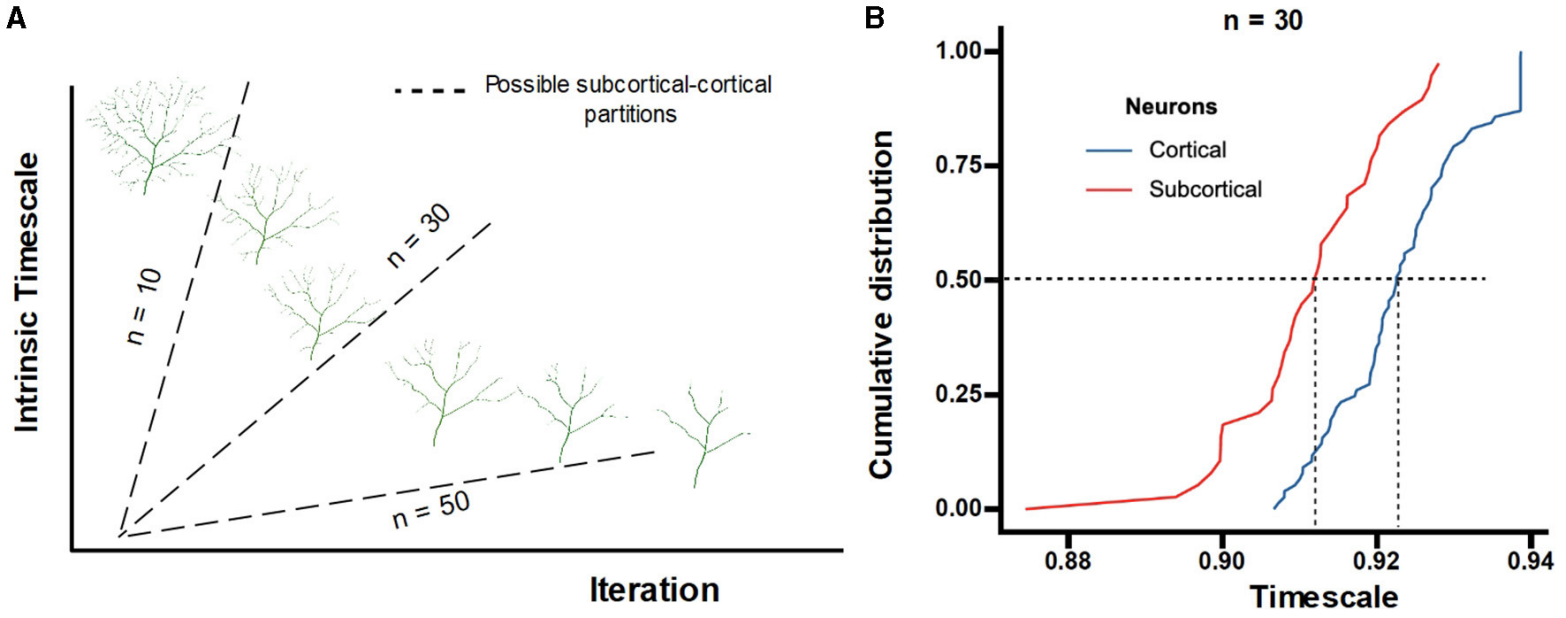
**(A)** Pruning iterations are used to divide the generated neurons into two groups: subcortical or cortical functional networks. **(B)** Distinction between subcortical and cortical functional networks for the number of pruning iterations *n* = 30.

Cytoarchitectonic studies demonstrate that the dendritic size decreases for increasing hierarchical levels (Hilgetag et al., 2019). Therefore, the pruning iteration reveals a gradient embodying the various hierarchical levels. Considering the spatial position of the subcortical and cortical network, the neurons generated at larger pruning iterations may represent neurons situated in brain regions higher up in the hierarchy. In the absence of a precise mapping relationship, we explore different possible neuron partitions for subcortical to cortical functional networks based on the number of iterations (as seen in Figure 8A). Under this assumption, the cumulative distribution of neuron INT demonstrates a clear hierarchical order of subcortical to cortical functional networks (Figure 8B). The cumulative distribution of neuron INT with different numbers of pruning iterations (n = 10 and n = 50) shows very similar results (Supplementary Figure 2). Statistical tests also substantiate the lower INT of subcortical networks compared to cortical networks (n = 10: *t*_*subcortical*_ = 3.239 *p* = 0.012; n = 30: *t*_*subcortical*_ = 3.451 *p* = 0.002; n = 50: *t*_*subcortical*_ = 4.639 *p* = 0.001 FDR-corrected). The results indicate that regardless of the partition used, a significant distinction between INT of neurons located at subcortical and cortical regions is observed.

## 4 Discussion

In this study, we examined intrinsic neural timescales (INT) across various functional networks spanning from subcortical to cortical regions, aiming to uncover their underlying neuronal mechanisms. Utilizing resting-state fMRI signals, functional networks were delineated, and the INT was derived from the autocorrelation functions. Notably, we observed a significant difference in intrinsic timescales, with subcortical networks exhibiting shorter INT values, indicating a gradient of INT along the cortical-subcortical axis. To better understand the contribution of dendritic size to this gradient of INT, we simulated neuronal structures across different anatomical levels using a pruning algorithm, motivated by cytoarchitectonic studies that show larger pyramidal neurons in regions located higher in the hierarchy (Beul and Hilgetag, 2019). Our findings revealed a correlation between shorter dendritic size, typical from higher anatomical levels, and longer INT. This result indicates that the gradient of intrinsic timescales across cortical and subcortical networks aligns with the gradient of INT observed at the neuronal level, highlighting the putative contribution of neuronal dendrites to the hierarchy of INT observed in functional brain networks.

### 4.1 Gradient of INT reflects a hierarchy of functions in the brain

Evidence supports a hierarchical organization of INT within regions of the brain (Hasson et al., 2015; Murray et al., 2014). Consistent with prior research, we reveal a hierarchy of INT in brain networks with shorter INT in the subcortical network (SC) and longer INT in the default mode (DMN), visual (VIS), sensorimotor (SMN), central executive (CC), and auditory (AUD) networks. Extending this hierarchical timescale beyond large-scale networks, we observe an INT gradient along cortical to subcortical networks, despite the spatial disparities between them.

INT refers to the duration over which neural information is stored within a local brain area (Chaudhuri et al., 2015; Deco et al., 2019; Farzan et al., 2017; Gollo et al., 2017, 2015). Hence, the length of INT reflects the hierarchy of functions in the brain (Cocchi et al., 2016; Hasson et al., 2015; Murray et al., 2014; Watanabe et al., 2019). The subcortical network, which includes regions such as the thalamus, caudate and putamen, shows the shortest intrinsic timescales. Given the function of exerting cognitive, primary affective that the subcortical network involves (Koshiyama et al., 2018; Johnson, 2005), this may reflect the rapid processing of sensory information and the coordination of basic physiological functions. In contrast, cortical networks, including networks such as the sensorimotor network and the central executive network, exhibit longer intrinsic timescales. Connecting the functions that cortical networks are associated with, the distinct cortical-subcortical INT substantiates that those cortical regions are involved in higher-order cognitive processes, which unfold over longer timescales (Funahashi, 2017; Jung et al., 2017).

The brain encompasses faster unimodal areas (e.g., the primary/secondary visual cortex and auditory cortex) and slower transmodal areas (e.g., lateral and medial prefrontal cortex) with varying processing lengths (Wolff et al., 2022). Consistent with this, we found that cortical networks have longer INT. Intriguing, the opposite pattern of the hierarchy of INT was observed in infancy (Truzzi and Cusack, 2023). This could be linked to brain function development. As the brain evolves and matures, cortical regions emerge later and undergo significant expansion (Bayne et al., 2023). Gradually engaging in higher-order cognitive functions such as reasoning, language, planning, and decision-making, these regions enhance their ability to integrate information, leading to an increase in INT. For example, the prefrontal cortex, which is one of the last regions to fully mature, occupies the top of the hierarchy and exhibits the longest INT. It remains unclear at which developmental stage the brain reaches a maturation of its hierarchy of timescales. Our study sample, composed of younger adults aged 19 to 22, indicates that at this stage, the brain has already established a hierarchy of INT.

A hierarchy of intrinsic timescales in the brain, driven by neuronal heterogeneity where neurons with shorter dendrites have longer timescales and brain regions at the bottom of the hierarchy have neurons with larger dendrites than those at the top, improves our understanding of the relationship between neuronal structure and brain function. Regions with higher neuron density (Beul and Hilgetag, 2019) support complex and rapid processing, while regions with fewer neurons but more complex dendritic morphology are involved in more integrative and prolonged tasks. This hierarchical organization allows for rapid, short-timescale processing in lower-order sensory regions with larger neurons, and slower, long-timescale processing in higher-order regions with smaller neurons, such as the default-mode network (DMN).

Our study also indicates a substantial gap between the timescales of neuronal spikes and fMRI signals from brain regions, highlighting that various elements at different levels of brain organization contribute to the hierarchy of time scales in the brain. This includes the density and morphology of dendritic spines (Cavanagh et al., 2020), which influence synaptic strength and temporal integration; cortical columns and layers (Mejias et al., 2016), which integrate local and large-scale processing; the connectivity patterns within neuronal circuits, which determine information flow and coordination; and the topology of brain networks (Gollo et al., 2015), which dictates hierarchical information processing. While we have begun to piece together some of these multiscale elements, considerable additional work is still needed to fully reveal how the brain efficiently processes information across multiple timescales and the intricate relationship between neuronal structure and brain function across the different levels of structure within the nervous system.

### 4.2 Possible explanations for the relationship between the dendritic size and INT

Neuronal dendrites play a crucial role in neuron function and information processing (London and Häusser, 2005). Progressive dendritic pruning, associated with neurodegeneration, has been linked to a decrease in the dynamic range, and a decrease in the energy consumption (Kirch and Gollo, 2021). Here we show that pruning reduces the neuronal firing rate, and these changes drive the observed increase of intrinsic timescales. We have also demonstrated a robust linear relationship between neuronal firing rate and intrinsic timescales for neurons regardless of their dendritic structure.

A larger dendritic size could be considered to have a longer intrinsic neural timescales, as larger dendrites could provide more opportunities for synaptic input, and the increased surface area of larger dendrites can enable more synaptic connections, affecting overall the neuronal function (Major et al., 2013; Zheng et al., 2024; Palmer, 2014). As a result, neurons with more dendritic spines could be more likely to integrate information over longer periods. However, this relationship is not straightforward and depends on various factors, including the specific neuronal circuit (e.g., longer timescales have stronger levels of local recurrent connections; Chaudhuri et al., 2015), neuronal and synaptic types (excitatory or inhibitory Torres-Gomez et al., 2020), and functional demands (Cavanagh et al., 2020). Furthermore, neurotransmitter receptor expressions were also considered important determinants of the neuron's timescales, considering their effects on the brain's persistent activity (Wang, 2001; Burt et al., 2018; Wang et al., 2013).

Using the pruning algorithm to adjust the dendritic size progression and obtaining the corresponding INT with simulated neuronal dynamics, we found that the dendritic size has a negative association with INT. This finding aligns with the cytoarchitectonic findings that higher anatomical levels have smaller dendritic arbors (Kiebel et al., 2008; Cocchi et al., 2016; D'Souza et al., 2016; Burt et al., 2018), and in higher levels of the hierarchy, information was processed across longer timescales (Himberger et al., 2018; Gollo et al., 2017). There are several possible explanations for this result. First, neurons with smaller dendritic trees may have fewer synaptic inputs and a more limited capacity for integrating incoming signals. As a result, it may take longer for these neurons to accumulate enough input to reach the threshold and generate an action potential (Zhang, 2019; Sengupta et al., 2010), leading to longer intrinsic timescales. Second, neurons with smaller dendritic trees may have higher input resistance, which can slow down the rate of membrane potential changes in response to synaptic inputs (Gulledge et al., 2005). This increased input resistance could contribute to longer timescales of neural activity and information processing. Third, the specific mechanisms underlying dendritic integration and computation may differ between neurons with smaller and larger dendritic trees. For example, smaller dendritic trees may exhibit more linear or passive integration properties, whereas larger dendritic trees may support more complex nonlinear computations (Koch et al., 1983; London and Häusser, 2005). These differences in dendritic processing, in conjunction with the differences in firing rate observed as a function of neuronal size could influence the overall timescale of neural activity.

### 4.3 Limitations and future consideration

The current research has some limitations for further consideration. First, we connect the simulated neurons with subcortical and cortical networks in the brain through cytoarchitectonic findings. Although the partitions of neurons do not affect the result of the cortex-to-subcortex hierarchy of INT, it would be informative to study INT of specific neurons found within the respective cortical and subcortical brain regions. Second, the association between the INT and offset reflects the trade-off between the short and long INT of the brain network. However, as an important parameter of the exponential fitting method, whether the offset varies significantly between networks or subcortical and cortical regions has yet to be explored. Finally, this study reveals an association between dendritic size and INT, while a more precise way of quantifying the relationship between them should be evaluated. It has been shown that INT is closely related to the strength and the pattern of neuron information encoding (Cavanagh et al., 2020; Wasmuht et al., 2018; Huang et al., 2023; Constantinidis et al., 2018; Lundqvist et al., 2018). Future studies could use the variation of the information encoding ability across neurons and tasks (decision-making; Constantinidis et al., 2018 or working memory; Lundqvist et al., 2018) to examine how dendritic size, density of spines (Cavanagh et al., 2020), and number of neurons in cortical regions (Beul and Hilgetag, 2019) affect their INT.

In addition, the fMRI data is only from 34 young adults and the set of prototype neurons comprises 13 neurons from 6 species. Future studies could incorporate a larger functional imaging dataset and could extend to a larger set of neurons within different regions of the same species. Furthermore, the neuronal dynamics considered here were simplified and modeled without considering the heterogeneity of ion channels due to the lack of data to inform the model. Though it simulates the dynamics of excitable systems, future studies could attempt more detailed biophysical models, for example, considering the membrane potential of each compartment as a continuous variable (Cuntz et al., 2021).

## 5 Conclusions

This study examined intrinsic neural timescales within cortical and subcortical functional networks and its interplay with neuronal dendrites. Employing resting-state fMRI signals, we computed the INT of these networks, uncovering a shorter INT in subcortical networks compared to cortical ones. Through the implementation of a pruning algorithm to simulate neurons across varying levels of integrity, we identified that regions with smaller dendritic sizes, typically found at higher hierarchical levels, exhibit longer INT. Furthermore, our investigation revealed that differences in INT at the neuronal level contribute to the variations of INT observed across distinct brain regions. These findings suggest that INT mirrors the functional hierarchy within the brain and that dendritic morphology exerts influence on the INT of brain functional networks. This study contributes to neuroscience by elucidating the neuronal mechanisms underlying INT and its association with brain structure and function. Additionally, it introduces an innovative approach for quantifying and simulating INT across different spatial scales. This study marks an initial advancement in our understanding of the intricate relationship between microscopic dendritic structure and the dynamic behavior of macroscopic functional networks.

## Data Availability

Publicly available datasets were analyzed in this study. The fMRI data can be found here: https://openneuro.org/datasets/ds003871/versions/1.0.2. The digital reconstructed neurons presented in study can be accessed via the following website: https://neuromorpho.org/.
