# Supplementary material for: Dendrites contribute to the gradient of intrinsic timescales encompassing cortical and subcortical brain networks
Source: Front Cell Neurosci. 2024 Sep 6;18:1404605. doi: 10.3389/fncel.2024.1404605 (PMC11412829; doi:10.3389/fncel.2024.1404605)
Supplement: Supplementary file 1 [file Data_Sheet_1.PDF]

Supplementary Data for "Neuronal Dendrites Contribute to The Gradient of Intrinsic Timescales Encompassing Cortical and Subcortical Brain Networks"

Table I. Peak coordinates of ICNs

ICNs	X	Y	Z
**Default-mode Domain: 16**			
Precuneus (9)	-0.5	-52.5	22.5
Precuneus (9)	-6.5	-73.5	10.5
Post cingulum cortex (30)	-0.5	-28.5	28.5
Anterior cingulum cortex (38)	-0.5	22.5	-13.5
Precuneus (43)	-0.5	-82.5	7.5
Post cingulum + Precuneus (47)	-0.5	-82.5	7.5
Angular gyrus (59)	20.5	-67.5	55.5
Angular gyrus (62)	47.5	-61.5	40.5
Precuneus (66)	-3.5	-64.5	-4.5
Cuneus+ Precuneus (74)	5.5	-79.5	43.5
Precuneus (80)	2.5	-70.5	43.5
Anterior cingulum cortex (87)	-0.5	13.5	31.5
Angular gyrus (88)	47.5	-61.5	40.5
Anterior cingulum cortex (90)	-0.5	-52.5	4.5
Precuneus (95)	-2.5	-49.5	7.5
Precuneus (98)	14.5	-49.5	-1.5
**Sensorimotor Domain: 6**			
Superior parietal lobule (8)	-24.5	-64.5	55.5
Paracentral lobule (33)	-23.5	-43.5	70.5
Postcentral gyrus (58)	-60.5	-25.5	31.5
Paracentral lobule (67)	-25.5	31.5	67..5
Paracentral lobule (68)	0.5	37.5	70.5
Superior parietal lobule (75)	-33.5	-58.5	58.5
Left precentral gyrus (79)	-35.5	-19.5	67.5
Superior parietal lobule (84)	-0.5	-58.5	64.5
**Visual Domain: 12**			
Middle occipital gyrus (5)	44.5	-64.5	-19.5
Middle temporal gyrus (34)	54.5	-58.5	-13.5
Left Lingual gyrus (17)	17.5	-67.5	-10.5
Cuneus (34)	5.5	-88.5	7.5
Middle occipital gyrus (60)	50.5	-67.5	1.5
Middle temporal gyrus (34)	56.5	-52.5	-13.5
Cuneus (82)	29.5	-91.5	1.5
Left Lingual gyrus (83)	-24	-46.5	-13.5
**Cognitive-control Domain: 18**			
Inferior frontal gyrus (20)	-50.5	16.5	31.5
Superior medial frontal gyrus (13)	-0.5	40.5	40.5
Middle cingulum (24)	-0.5	-7.5	46.5
Superior frontal gyrus (25)	-27.5	13.5	61.5
Middle cingulum (26)	-0.5	-13.5	37.5
Middle frontal gyrus (32)	-0.5	-10.5	61.5
Supplementary motor area (42)	-30.5	1.5	67.5
Supplementary motor area (49)	-0.5	10.5	61.5
Right Inferior parietal lobule (51)	53.5	-43.5	49.5
Middle frontal gyrus (52)	-51.5	19.5	-7.5
Supplementary motor area (70)	-21.5	-25.5	70.5
Insula (76)	-39.5	16.5	-10.5
Inferior frontal gyrus (85)	-54.5	13.5	22.5
Inferior frontal gyrus (86)	-57.5	-4.5	-1.5
Superior medial frontal gyrus (93)	-0.5	34.5	58.5
Insula (94)	-44.5	-10.5	-1.5
**Sub-cortical Domain: 7**			
Caudate (20)	-24.5	1.5	-7.5
Posterior thalamus (21)	-0.5	-22.5	-4.5
Caudate (23)	-21.5	-19.5	-16.5
Thalamus (41)	-0.5	-4.5	-10.5
Thalamus (44)	-0.5	-22.5	-7.5
Thalamus (53)	-0.5	-28.5	-4.5
Thalamus (55)	-0.5	-1.5	-16.5

Thalamus (71)	-18.5	-37.5	1.5
Caudate (40)	-0.5	7.5	1.5
Putamen (49)	-5.5	-1.5	-10.5
**Auditory Domain: 6**			
Right Superior temporal gyrus (6)	-57.5	-43.5	1.5
Superior temporal gyrus (15)	-48.5	-25.5	-10.5
Superior temporal gyrus (63)	-63.5	-14.5	2.5
Superior temporal gyrus (81)	56.5	31.5	-7.5

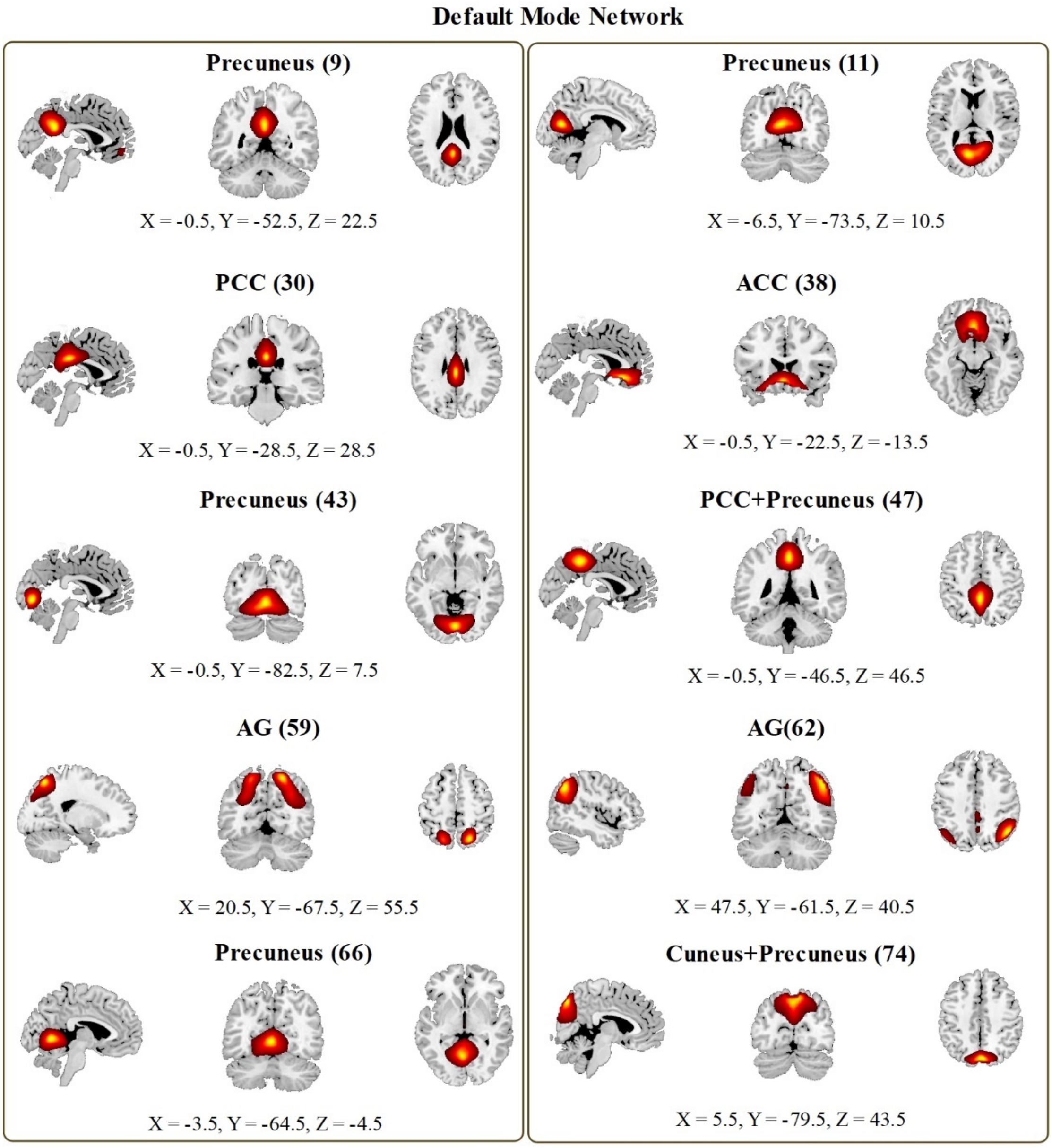
Default Mode Network
Precuneus (9)
X = -0.5, Y = -52.5, Z = 22.5
Precuneus (11)
X = -6.5, Y = -73.5, Z = 10.5
PCC (30)
X = -0.5, Y = -28.5, Z = 28.5
ACC (38)
X = -0.5, Y = -22.5, Z = -13.5
Precuneus (43)
X = -0.5, Y = -82.5, Z = 7.5
PCC+Precuneus (47)
X = -0.5, Y = -46.5, Z = 46.5
AG (59)
X = 20.5, Y = -67.5, Z = 55.5
AG(62)
X = 47.5, Y = -61.5, Z = 40.5
Precuneus (66)
X = -3.5, Y = -64.5, Z = -4.5
Cuneus+Precuneus (74)
X = 5.5, Y = -79.5, Z = 43.5

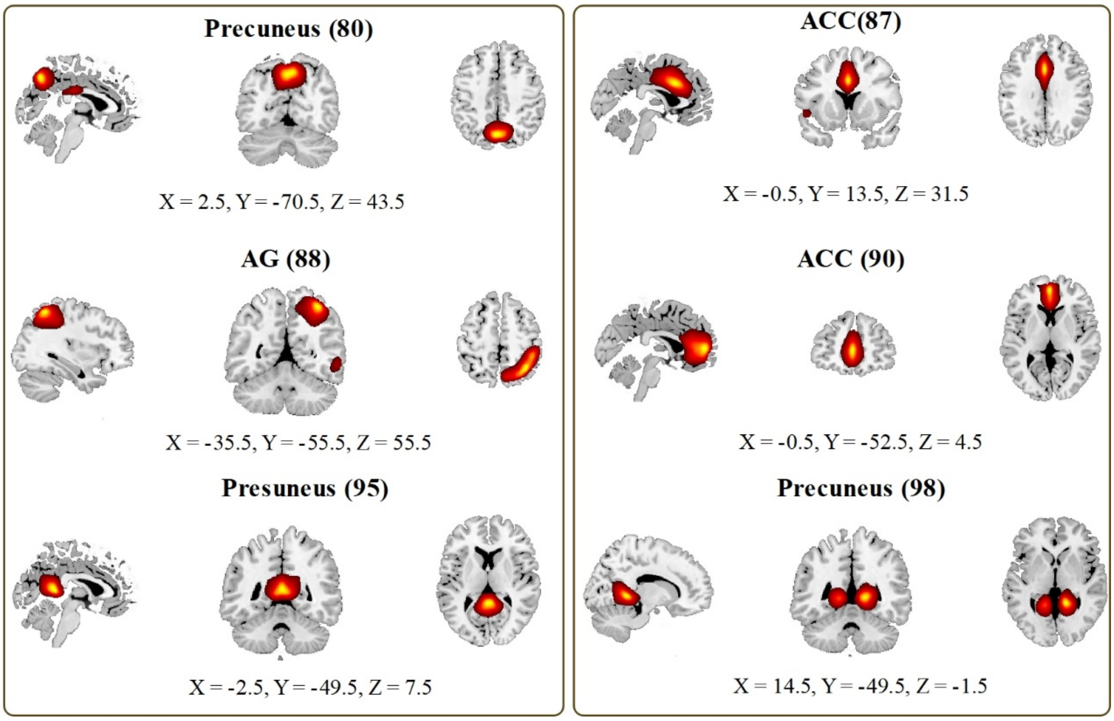

Figure 1-1. Spatial maps of intrinsic connectivity networks in default mode domain. Sagittal, coronal, and axial slices are shown at the maximal t-statistic for clusters larger than 3 cm^3.

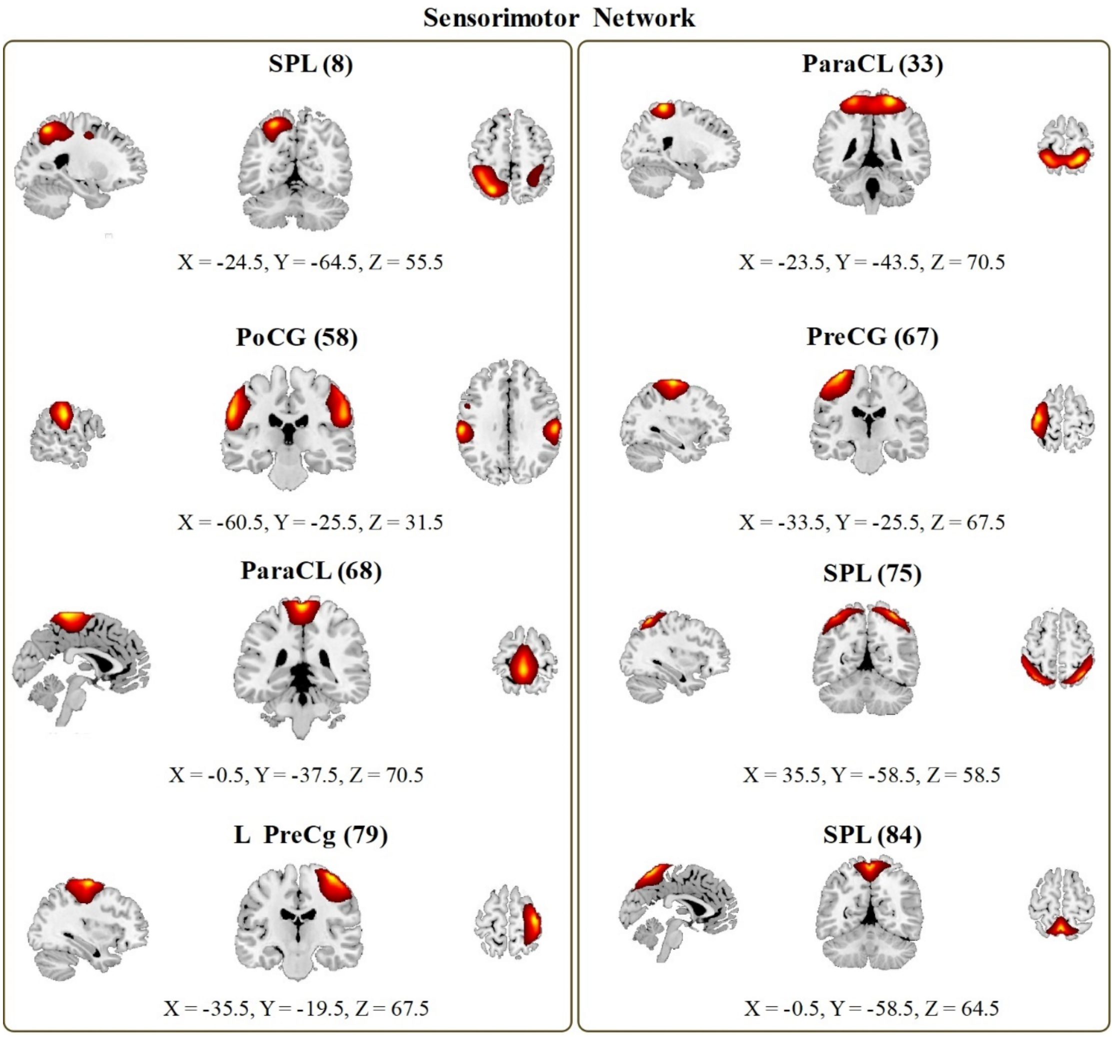

Figure 1-2. Spatial maps of intrinsic connectivity networks in sensorimotor domain. Sagittal, coronal, and axial slices are shown at the maximal t-statistic for clusters larger than 3 cm^3.

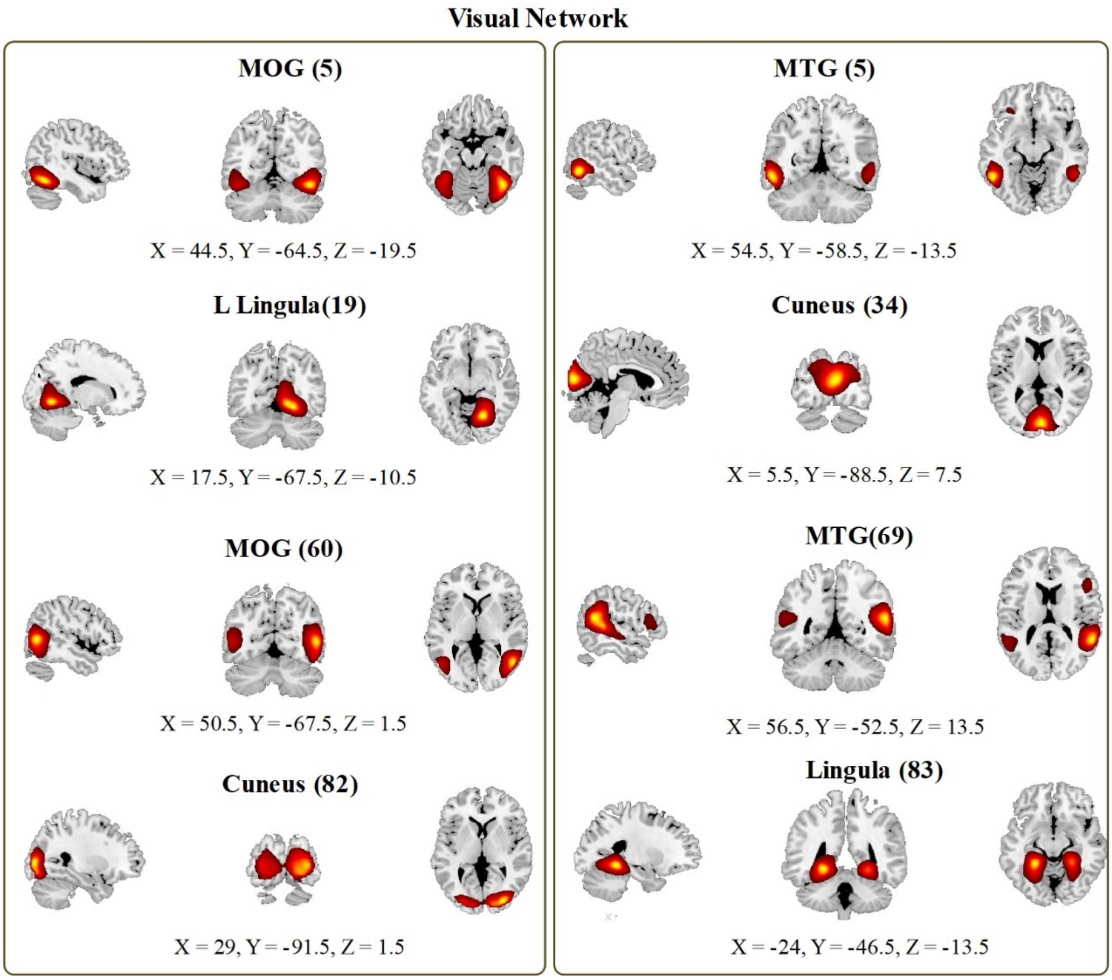

Figure 1-3. Spatial maps of intrinsic connectivity networks in visual domain. Sagittal, coronal, and axial slices are shown at the maximal t-statistic for clusters larger than 3 cm^3.

Cognitive Control Network

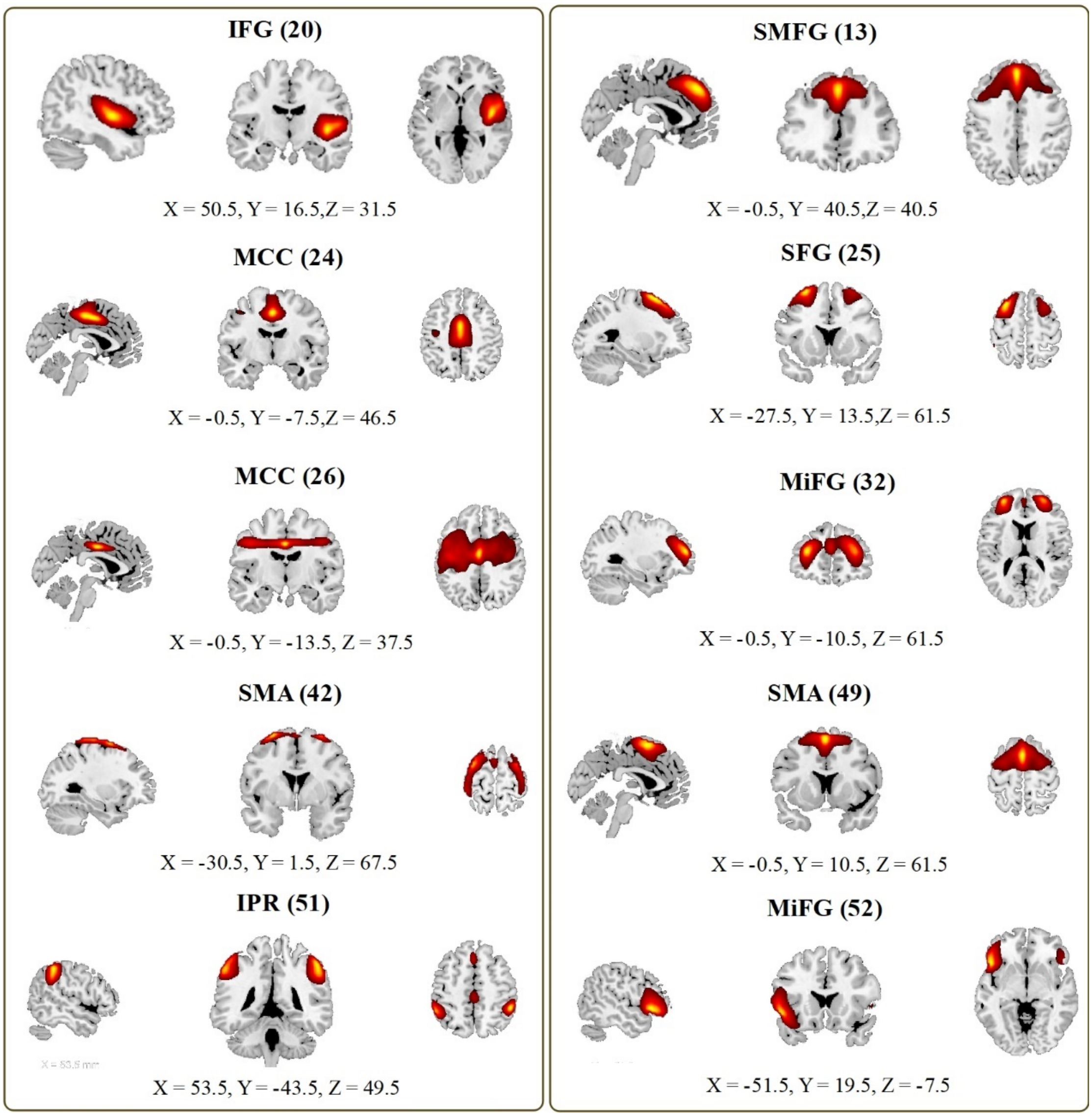

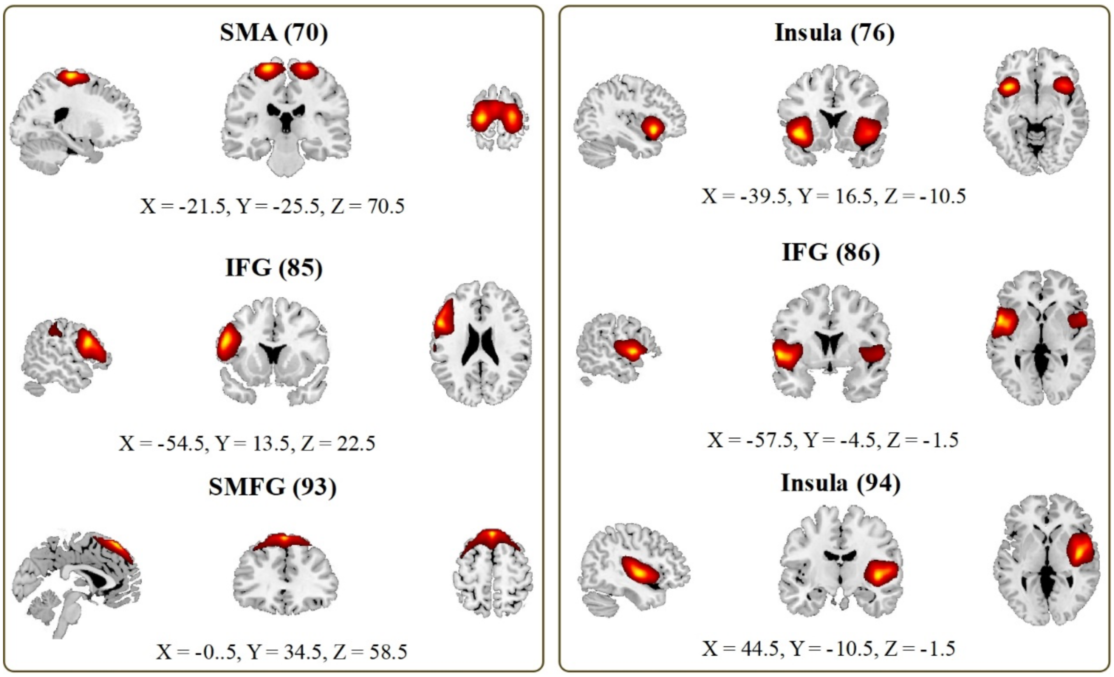

Figure 1-4. Spatial maps of intrinsic connectivity networks in cognitive control domain. Sagittal, coronal, and axial slices are shown at the maximal t-statistic for clusters larger than 3 cm^3.

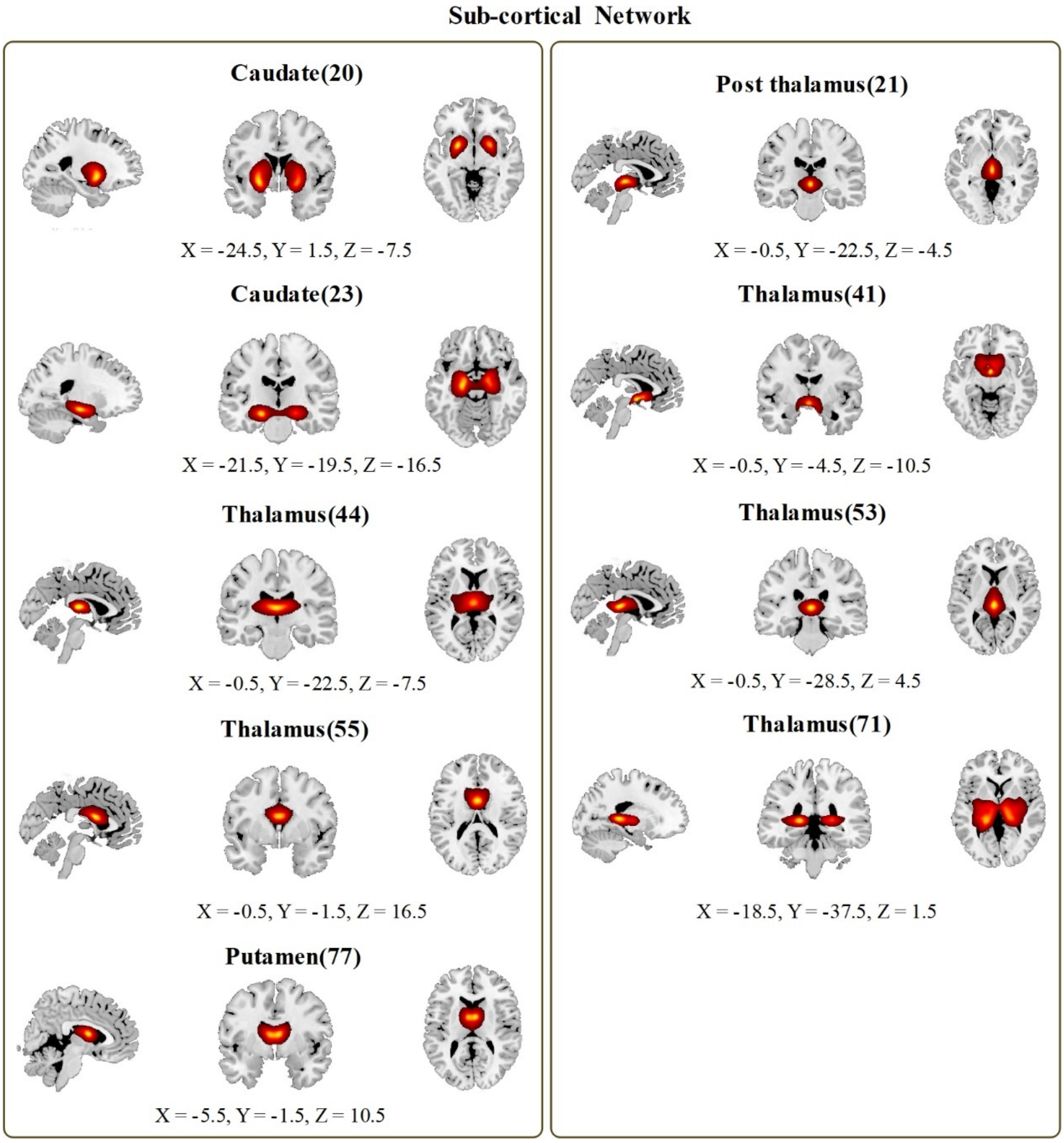

Figure 1-5. Spatial maps of intrinsic connectivity networks in subcortical domain. Sagittal, coronal, and axial slices are shown at the maximal t-statistic for clusters larger than 3 cm^3.

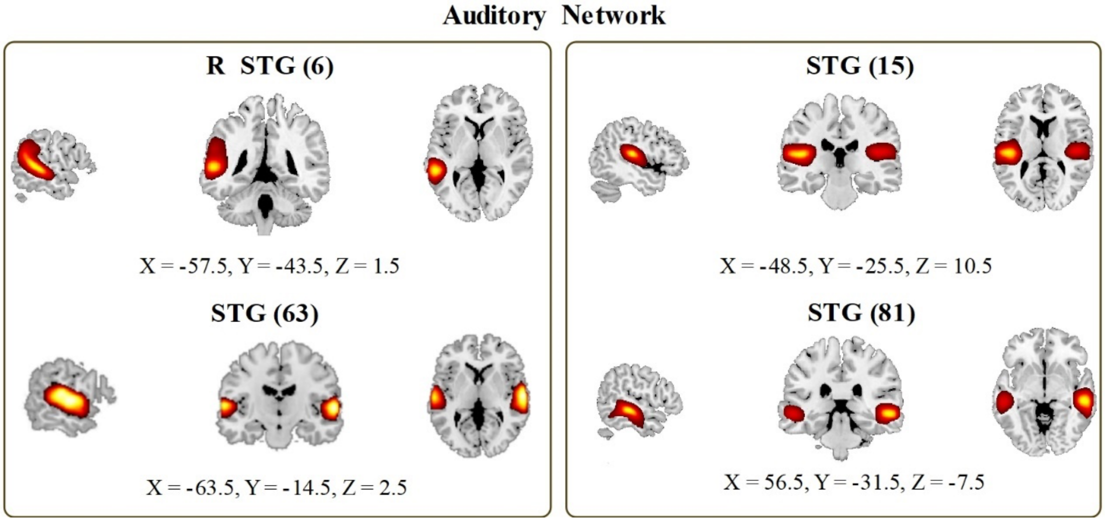

Figure 1-6. Spatial maps of intrinsic connectivity networks in subcortical domain. Sagittal, coronal, and axial slices are shown at the maximal t-statistic for clusters larger than 3 cm^3.

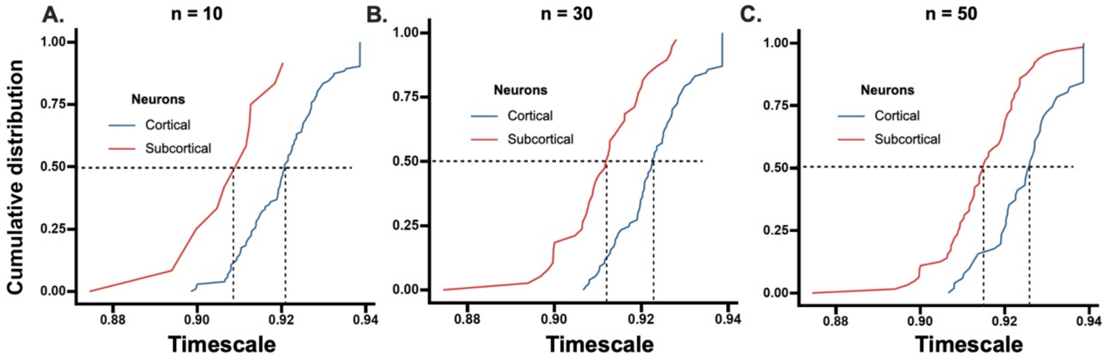

Figure 2. The pruning iterations, n = 10, 30, 50, are used to divide the produced neurons into subcortical to cortical function networks. Result shows, though with different partitions, the significant distinct subcortical to cortical function network can be observed.
